# Heterogeneous courses of obsessive–compulsive disorders—better data on a lifetime perspective urgently needed

**DOI:** 10.1007/s00787-022-02043-1

**Published:** 2022-07-13

**Authors:** Veit Roessner, Stefan Ehrlich, Lea Backhausen, Sarah Rempel, Anne Uhlmann

**Affiliations:** 1 Department of Child and Adolescent Psychiatry, Faculty of Medicine, University Hospital Carl Gustav Carus, TU Dresden, Fetscherstrasse 74, 01307 Dresden, Germany; 2 Division of Psychological and Social Medicine and Developmental Neurosciences, Faculty of Medicine, TU Dresden, Fetscherstrasse 74, 01307 Dresden, Germany

In the present issue of ECAP, Vos et al. [1] rightly criticize that the main focus of most longitudinal studies about considerable differences in presence and severity of attention-deficit/hyperactivity disorder (ADHD) symptoms is about the period from childhood to early adolescence. “This leaves important gaps of knowledge in our understanding of symptom change beyond these ages”. This is all the more regrettable since better knowledge about the heterogeneous courses of ADHD symptoms is critically needed to better inform patients, relatives, and clinicians regarding the individuals at risk for unfavourable outcomes in later life [2]. Thus, their study had two aims: first, to describe the heterogeneous courses of ADHD, and second, to identify potential differentiating characteristics of these ADHD symptom courses beyond middle childhood into early adulthood. Using multilevel multinomial logistic regression, they identified a seven-class solution reflecting highly heterogeneous courses of ADHD, with stable, decreasing, and increasing trajectories of inattention and hyperactivity-impulsivity.

This criticism of insufficient knowledge about quantitative and qualitative details of different courses and prognosis of ADHD is fully understandable. However, in another very prevalent mental disorder, obsessive–compulsive disorder (OCD) with a lifetime prevalence of 2–3%, such knowledge is even more limited. Although OCD ranks among the top 10 causes of disability worldwide [3], a current (23/06/2022) search for scientific publications in PubMed returned only about 11,000 hits for OCD compared to 45,000 hits for ADHD.

In our opinion, there is a need for even more comprehensive research in OCD to reach the same level of evidence on prevalence rates and courses (i.e. illness trajectories) as in ADHD, because there are obvious differences between ADHD and OCD resulting in “the much more frayed psychopathological edges” in OCD. The latter are reflected, for example, in the term “obsessive–compulsive spectrum” (PubMed *n* = 410 hits) while the term “attention-deficit spectrum” is virtually non-existent (PubMed *n* = 2 hits).

Nevertheless, in both fields of ADHD and OCD research, there is a long and lively discussion about distinction from commonly co-existing disorders as well as differentiation between disorder-inherent vs. co-existing, non-core symptoms. For example, in ADHD the role of irritability (= symptom) [4] or adolescent depression (= disorder) [5] is under investigation. Similarly, in OCD, there is much discussion about psychopathological commonalities and discrepancies, e.g., hoarding (= symptom of OCD or co-existing disorder) in children and adolescents with OCD [6], and about overlap or co-existence of autism/autistic traits and OCD [7]. In this vein, Duholm et al. [8] explore the potential clinical role of health anxiety (HA) symptoms in children and adolescents with OCD in the present issue of ECAP. HA symptoms were present in 31% of the study participants. These patients had also more other anxiety symptoms and co-existing anxiety disorders as well as significantly more types of OCD symptoms. Fortunately, they responded to exposure-based cognitive behavioural therapy (CBT) similarly to those without HA symptoms.

“The much more frayed psychopathological edges” in OCD are also reflected by the lively debate about the prevalence, overlap, co-existence, etc. of obsessive–compulsive personality disorder (OCPD) e.g. in patients with obsessive–compulsive disorder (OCD) (for a systematic review and meta-analysis see [9]), complicating diagnosis and therefore research studies, while there is no ADHD-related personality disorder.

While in ADHD symptom constellations (e.g. impulsive/hyperactive subtype, inattentive and distractible subtype) as subtype defining factors have been in the centre of discussion for a long time, the discussion about subtypes in OCD is more closely related to possible, clearly distinguishable courses of OCD symptoms (e.g. early-onset vs. late-onset OCD). Accordingly, we are not aware of any discussion about a bimodal distribution of age of onset in ADHD as it is the case for OCD [10], although late-onset ADHD has been studied extensively in the past few years and the age of first onset of symptoms vs. first diagnosis of ADHD as well as of OCD is under debate [11, 12].

The bimodal distribution of age of onset in OCD is commonly reported with about 11 years of age for early-onset OCD vs. about 19–23 years of age for late-onset OCD. However, as so often in clinical research, there are conflicting results and this has contributed to the decision not to recommend age of onset as an OCD subtype in DSM-5. One source for this heterogeneity of findings is the much too seldom discussed delay between mean age of onset and age at diagnosis of OCD. For example, Hezel et al. [13] recently reported that the mean age of onset was 13.6 years and age at diagnosis was 20.7 years old, resulting in a mean delay of 7.1 years. Unfortunately, an association between earlier onset of OC symptoms and greater delay to diagnosis has been found. One reason for the delay might be that individuals with early-onset of OC symptoms tend to wait longer before seeking treatment than those who experience symptom onset after childhood or adolescence, which likely contributes to a delay to receiving a proper diagnosis. Stengler et al. [14] wrote that “early, still unspecific occurrences are either trivialized or normalized by mistake by the persons affected themselves, but also by family members, often the person’s affected parents” (p. 817).

To improve and refine diagnosis and subsequent treatment, research on not only course-related but also aetiology-related subtypes could be helpful. There is increasing evidence that patients with early-onset compared to late-onset as well as tic-related vs. non-tic-related OCD are likely to have a stronger genetic or biological component [15]. Interestingly, while no course-related specifier was included in DSM-5, the base of evidence for a tic-related (i.e. the individual has a current or past history of a tic disorder) specifier has been judged broader [16]. However, it remains unclear how large the overlap of early-onset OCD with tic-related OCD is.

In summary, the mentioned heterogeneity “around OCD” together with the findings that 39% of mental health professionals and 50.5% of primary care physicians misdiagnosed OCD when asked to give their diagnostic impressions of individuals presented in clinical vignettes, and that OCD is frequently undetected even in clinical settings, highlights the need for more naturalistic, longitudinal, high-quality studies on the onset and courses of OC symptoms to guide clinicians much better and thoroughly [13].

This is all the more important, as research in OCD suggests that early intervention and long-term treatment result in both better symptomatic and functional outcomes [17], whereas in ADHD, the effect of treatment on symptomatic outcome, i.e., reduction of the core symptoms seems to be weaker.

Independent of the generation of new, more detailed and higher quality findings on different courses and prognosis of OCD, there is still evidence and consensus that increasing awareness of OC symptoms in families with small children and among paediatricians is the most important challenge to continuously tackle [13].
